# Effect of *Acheta domesticus* Powder Incorporation on Nutritional Composition, Technological Properties, and Sensory Acceptance of Wheat Bread

**DOI:** 10.3390/insects16090972

**Published:** 2025-09-17

**Authors:** Agnieszka Orkusz, Martyna Orkusz

**Affiliations:** 1 Department of Biotechnology and Food Analysis, Wroclaw University of Economics and Business, 53-345 Wroclaw, Poland; 2 Faculty of Biotechnology and Food Science, Wroclaw University of Environmental and Life Sciences, 50-375 Wroclaw, Poland; 125360@student.upwr.edu.pl

**Keywords:** wheat bread, edible insects, *Acheta domesticus*, bread fortification, consumer acceptance, technological properties

## Abstract

The fortification of bakery products with alternative protein sources, including edible insects, offers a promising approach to improving nutritional quality while addressing sustainability challenges. This study evaluated graded replacement of type 750 wheat flour with *Acheta domesticus* (house cricket) powder—together with an extreme 100% cricket-powder formulation—on the nutritional composition, color, particle size distribution, fermentative properties, baking loss, crumb hardness, and sensory quality of bread. Fifteen baked variants were prepared: a 100% wheat flour control; thirteen wheat–cricket blends containing 5–90% cricket powder; and an extreme formulation with 100% cricket powder. Increasing cricket-powder levels significantly increased protein, fat, fiber, zinc, and riboflavin contents while decreasing carbohydrate and starch levels. Technologically, higher substitution levels resulted in darker crumb color, a shift toward coarser particle size distribution, reduced gas retention during proofing, and increased baking loss. Sensory analysis indicated that up to 15% inclusion maintained full consumer acceptability, while 20–25% was at the acceptance threshold. Above 35%, acceptability declined sharply due to intensified earthy flavors and textural changes. The findings highlight 15% inclusion as the optimal balance between enhanced nutritional value and sensory quality, with potential for higher incorporation if appropriate technological modifications are applied.

## Simple Summary

Bread is one of the most widely consumed foods worldwide, and enriching it with alternative protein sources is a challenge in modern food technology. This study prepared fifteen bread variants: one made entirely from wheat flour, one made entirely from cricket powder (*Acheta domesticus*), and thirteen blends containing 5% to 90% cricket powder. The following analyses were carried out: color measurement of the flour blends, particle size distribution, dough fermentation properties, nutritional value (including Nutritional Quality Index), baking loss, crumb hardness, and consumer sensory evaluation, including taste, aroma, hardness, chewiness, gumminess, and overall acceptability. The analysis determined the effect of different levels of cricket powder on the technological and nutritional properties of bread, as well as its consumer acceptance. This is likely the first study to apply such a wide range of insect powder additions—from low to very high levels—combined with a comprehensive assessment of physicochemical, nutritional, and sensory parameters, providing knowledge essential for developing breads enriched with insect-derived protein.

## 1. Introduction

Food enrichment and fortification are essential trends in modern food technology, aimed at enhancing nutritional value, increasing health-promoting potential, and improving techno-functional properties. The success of such initiatives largely depends on consumer acceptance and the level of consumption of a given product. Due to their widespread presence in the daily diet and broad acceptance, bakery products—such as bread and pasta—are particularly suitable carriers for enriching ingredients, allowing direct fortification during processing [1,2,3,4,5,6,7,8]. Such fortification can improve nutritional value as well as functional and sensory characteristics. For example, the nutritional quality of bread can be enhanced by adding legumes, oilseeds, or herbs [9].

Increasingly, there is a shift towards unconventional nutrient sources and bioactive ingredients, notably from edible insects, which are rich in protein and micronutrients and offer environmental advantages due to their high feed conversion efficiency and ability to thrive on by-products and cereal grains [10,11,12].

Ethically, using insect-based products is associated with sustainability goals, including reduced greenhouse gas emissions, lower land and water requirements, and improved animal welfare compared with conventional livestock production. These aspects are underscored in the European Union’s “Farm to Fork” Strategy, which aims to expedite the transformation toward a food system grounded in fairness, public health, and environmental sustainability [13].

Crickets (Orthoptera: Gryllidae), particularly the house cricket (*Acheta domesticus*), which is commercially available worldwide, are among the most widely used species in the food industry and attract considerable scientific attention.

At the regulatory level, *Acheta domesticus* and its processed forms are classified as novel foods under Regulation (EU) 2015/2283 [14]. Following EFSA’s safety assessments [15], the European Commission authorized frozen, dried, and powdered forms of *Acheta domesticus* (Commission Implementing Regulation (EU) 2022/188 [16]). For bakery products such as bread and rolls, the maximum permitted level of cricket powder is 10 g/100 g of the final product, while for the frozen form, a higher level of 30 g/100 g is allowed. These limits were established based on EFSA’s toxicological, nutritional, and allergenic evaluations [15]. They are designed to ensure consumer safety and preserve the food matrix’s technological functionality. Importantly, such products are intended for the general population. However, EFSA highlighted that individuals allergic to crustaceans, mollusks, and dust mites may be at risk of cross-reactive allergic responses. Therefore, mandatory labeling is required to protect sensitive groups.

Since bread is one of the main products considered for fortification, it is essential to look more closely at the properties of wheat flour (WF), the key ingredient in its production. Wheat flour bread is one of the most widespread bread species globally, constituting an essential part of the human diet. Gluten in wheat flour imparts viscoelastic properties to the dough, enabling it to retain the carbon dioxide generated during proofing, significantly influencing the appearance, structure, and texture of bread [17,18,19].

Type 750 wheat flour contains sufficient gluten for bread making and other baked goods that require a good dough structure. Its wet gluten content of 30.0–31.9 g is typical for bread flour, and its high elasticity facilitates the development of a stable gluten network, supporting the formation of a well-aerated crumb structure [19,20,21].

Incorporating insect flour into wheat bread introduces an unconventional raw material with a high protein and micronutrient content, which may affect not only the nutritional value of the bread but also its sensory attributes and texture [11,22]. Therefore, determining the optimal level of addition that does not compromise sensory quality or physical properties is essential. In this context, the present study evaluated the effect of *Acheta domesticus* powder on the nutritional composition, physicochemical parameters, technological properties, and sensory quality of wheat bread, aiming to identify the addition level that ensures high consumer acceptability while maintaining desirable bread structure and texture. Unlike previous research, which typically examined low to moderate inclusion levels of *Acheta domesticus* powder in bread (up to 10–20%) [23,24,25], this study systematically investigated a broad substitution range from 5% to 90%, as well as an extreme 100% cricket-powder formulation. This comprehensive approach enabled the identification of an optimal inclusion level and provided unprecedented insight into consumer responses and bread-making performance at very high insect powder levels, which had not been reported previously.

## 2. Materials and Methods

### 2.1. Raw Materials

Wheat bread flour type 750 was provided by Grain and Milling Company “PZZ” in Stoisław S.A. (Stoisław, Poland) and its composition per 100 g of product, as reported on the label, was as follows: 1.8 g of fats, of which 0.4 g were saturated fatty acids, 68.0 g of carbohydrates, of which 0.5 g were sugars, 12.0 g of proteins, 2.9 g of fiber, and 0.01 g of salt.

The cricket flour used in this study was obtained from a commercial supplier (SENS Foods, London, UK). According to the manufacturer’s description, it consists of 100% milled crickets—no roasting or baking is involved—and is produced using advanced drying methods that preserve nutrients. Dried house crickets (*Acheta domesticus*) in powder form contained 20 g of fats (of which 5.2 g were saturated fatty acids), 0.5 g of carbohydrates, 70 g of proteins, 9.5 g of fiber, and 0.8 g of salt per 100 g. All raw materials were stored in sealed containers under dry and refrigerated conditions until further use.

### 2.2. Preparation of the Binary Blends

Mixtures of cricket powder (CP) and wheat flour (WF) were prepared in the following proportions: 5%, 10%, 15%, 20%, 25%, 30%, 35%, 40%, 50%, 60%, 70%, 80%, and 90% CP, with the corresponding amounts of 95%, 90%, 85%, 80%, 75%, 70%, 65%, 60%, 50%, 40%, 30%, 20%, and 10% WF. The mixtures were labeled as M1–M13, respectively, and prepared in a rotary drum mixer (TM100, zu Jeddeloh GmbH, Winsen, Germany, art. no. 87290910) operated for 10 min.

Wheat flour (100%) was used as the control sample. Additionally, 100% *Acheta domesticus* powder was analyzed as an extreme variant for comparison.

### 2.3. Colorimetric Parameters

The color parameters lightness (L*), redness (a*), and yellowness (b*) of wheat flour, *Acheta domesticus* powder, and their mixtures were measured using a chroma meter (CR-310, Konica Minolta, Ramsey, NJ, USA) according to the official method CIELab.

The color measurement was carried out on a Petri dish with a diameter of 100 mm. The measurement was performed three times at different locations on the dish.

### 2.4. Granulometric Distribution

The granulometric distribution of the tested flours and flour blends was determined using a set of stainless steel laboratory sieves (W.S. Tyler, Mentor, OH, USA) with nominal mesh sizes of 500 µm, 180 µm, and 125 µm, together with a metal receiver and cover. Before the analysis, each sieve and the receiver were weighed on an electronic balance (Radwag WPS 210/C/2; Radom, Poland, readability 0.001 g), and the masses were recorded. A 100 g portion of the sample was placed on the uppermost sieve. The sieves were assembled in descending order of mesh size, covered, and mounted on an electric laboratory sieve shaker (AS 200, Retsch GmbH, Haan, Germany), which was operated for 10 min at an amplitude of 1.5 mm and a frequency of 60 Hz. Upon completion of the sieving process, the material retained on each sieve and in the receiver was weighed, and the proportion of each fraction relative to the total sample mass was calculated. The measurement was performed three times for each sample.

### 2.5. Assessment of Fermentative Properties

Fermentative properties of the flours were assessed using baker’s yeast (*Saccharomyces cerevisiae*, Dr. Oetker, Gdańsk, Poland) in three replicates for each tested flour. A 100 g portion of flour was weighed on an analytical balance (Radwag XA 110/2X; Radom, Poland, readability 0.1 mg) and mixed with 1.5 g of salt, 5 g of sugar, and 6 mL of rapeseed oil. The yeast (3 g) was dissolved in 100 mL of deionized water at 25 °C and added to the dry mixture, after which the dough was mixed for approximately 5 min with a stainless steel spoon in a 1 L glass beaker (Simax, Singapore).

A 50 mL portion of the dough was transferred into each of three 250 mL graduated cylinders (graduated to 5 mL), the surface was leveled with a spatula, and the cylinders were covered with foam disks.

The cylinders were placed in a compact incubator (Labnet I-5110A; Labnet International, Iselin, NJ, USA) that maintained a constant temperature of 30 ± 0.6 °C and relative humidity of approximately 65%. The incubator chamber had a thermometer port, which enabled temperature monitoring without opening the door. The dough volume was read through the incubator window every 10 min until the dough collapsed. The maximum dough volume and the time required to reach it were recorded.

### 2.6. Analysis of the Baking Properties

#### 2.6.1. Bread Preparation

The breads were prepared in fifteen variants: one prepared exclusively with wheat flour (control), one prepared exclusively with insect powder (*Acheta domesticus*), and thirteen variants consisting of mixtures of wheat flour and insect powder at different levels. The recipe included commonly used ingredients: oil, salt, sugar, dried yeast (Saccharomyces cerevisiae), and water, combined with the appropriate amounts of wheat flour and insect powder according to the experimental design. The specific compositions of all bread variants are presented in Table 1.

The dough ingredients were mixed mechanically using a kitchen mixer (Bosch MUMS2TW01; Bosch, Germany; 700 W) for 10 min. After mixing, the dough was divided and placed into pre-weighed metal baking tins. Subsequently, the dough was fermented and proofed in a proofing chamber (model 823HO; Bartscher GmbH, Salzkotten, Germany) at 30 °C and approximately 75% relative humidity for 30 min. The tins with dough were weighed before and after baking. Baking was carried out in a convection-steam oven (model 227804, Hendi Food Service Equipment, De Klomp, The Netherlands) at 180 °C for 20 min. After 30 min of cooling at room temperature, the baked loaves were removed from the tins and weighed. Instrumental and sensory evaluations were performed two hours after complete cooling of the samples. Three replicates were prepared for each determination, and the results were reported as mean values of the three measurements.

#### 2.6.2. Baking Loss

The baking loss was calculated from the difference between the dough mass and the baked product mass [26,27], according to the following formula, where

MD—dough mass [g],

MB—baked product mass after cooling [g].

$$BL\;[\%]=\frac{\left(MD-MB\right)\times 100\%}{MD}$$

#### 2.6.3. Crumb Hardness Assessment

Cylindrical samples (20 mm in height and 20 mm in diameter) were cut from the cross-sections of the bread using a cork borer. The samples were placed under the probe of a penetrometer (PCE-PTR 200N, PCE Instruments, Meschede, Germany) equipped with a polyacrylic measuring tip. The probe was lowered, and after release, the penetration depth (in mm) of the probe into the crumb under a constant load over 5 s was recorded. The greater the value in mm, the lower the hardness of the sample. For each sample, three independent measurements were performed.

The baked products were documented photographically using a digital camera (EOS 700D, Canon Inc., Tokyo, Japan), and the crumb cross-section was scanned with a flatbed scanner (Perfection V600, Seiko Epson Corp, Suwa, Japan).

### 2.7. Sensory Assessment

The sensory analysis of the obtained baked products was carried out as a semi-consumer assessment. Twenty-five untrained university students (14 females, 11 males, aged 22–24 years) participated in the evaluation. None of them had previous experience in sensory evaluation of food or insect-based products, and therefore, they are classified as non-expert assessors. The chosen panel size is consistent with semi-consumer tests, which provide indicative results for preliminary product development, as opposed to full consumer studies requiring ≥100 respondents.

The evaluation was based on a 9-point hedonic scale according to ISO 4121 [28], with boundary terms in Table 2.

Five sensory attributes (flavor, aroma, hardness, chewiness, gumminess) and the overall score were evaluated and compared with the control bread (100% wheat flour). To ensure correct understanding, the participants were provided with short definitions of chewiness and gumminess before the evaluation. The analysis was performed in a white-light laboratory meeting the requirements of ISO 8589 [29]. Samples (10 mm crumb cubes) were coded and presented randomly to minimize bias [30].

### 2.8. Nutritional Value

Basic data on the nutritional value of powdered *Acheta domesticus* and type 750 wheat flour were obtained from the product package labels. The vitamin and mineral content of the insects was supplemented based on literature data [31]. At the same time, the composition of the wheat flour was determined using the licensed computer program Diet 6D, which contains tables of the composition and nutritional values of food products [32]. The Diet 6D program was developed in 2018 at the Independent Laboratory of Epidemiology and Nutrition Standards, Institute of Food and Nutrition, Warsaw, Poland.

### 2.9. Nutritional Quality Index

The nutritional quality index (INQ), which serves as an indicator of food nutrient density, was computed for various nutrients employing the subsequent formula [33]:

$$INQ=\frac{ingredient\;content\;per\;100\;g\;of\;product\;\times \;energy\;requirement\;standard}{energy\;value\;per\;100g\;of\;product\;\times \;requirement\;standard\;for\;ingredient}$$

The INQ values for protein, as well as selected minerals (Na, K, Ca, P, Mg, Fe, Zn, Cu, Mn), and vitamins (A, E, B_1_, B_2_, B_3_, C) were determined by the nutritional requirements specified for a premenopausal female aged between 30 and 59 years, with a body weight of 59.9 kg, and engaged in moderate physical activity. The values of the daily recommended intake of nutrients come from the Polish standards developed in 2024 by the experts of the National Institute of Public Health–National Institute of Hygiene [34]. The calculations were executed utilizing a Microsoft Excel spreadsheet.

### 2.10. Statistical Analysis

All the analyses were performed in triplicate. The results are reported as means ± standard deviations (SD). Differences among mean values were analyzed by one-way analysis of variance (ANOVA) using the Statistica 13.0 package [35]. Tukey test was carried out to determine statistically significant differences (*p* < 0.05). Pearson’s correlation coefficients (r) were calculated to assess relationships between sensory attributes.

### 2.11. Ethical Statement

All participants gave informed consent for inclusion before participating in the study. The study was conducted in accordance with the Declaration of Helsinki, and the protocol was approved by the Research Ethics Committee of Wroclaw University of Economics and Business 43/2021.

## 3. Results and Discussion

### 3.1. Color Parameters

Color is widely recognized as one of the primary sensory attributes influencing consumer perception and product choice [36,37]. The substitution of wheat flour with increasing cricket powder levels significantly affected the colorimetric parameters L*, a*, and b* (Table 3).

The L* values, representing lightness, decreased progressively with increasing CP content, from 96.83 in 100% wheat flour to 51.92 in 100% cricket powder (Table 3). This corresponds to a 46.4% reduction in brightness, with a statistically significant decrease already observed at 5% CP (89.24).

The a* values increased from −0.07 in the sample containing 100% wheat flour (a negative value indicating a shift toward the green area) to 2.60 in the sample with 100% cricket powder (a positive value indicating a shift toward red) (Table 3). A marked increase in a* was already observed at the 5% CP level. The difference between the minimum and maximum values was 2.67 units, corresponding to a rise of over 2800%.

The b* values, which reflect the yellow component of color, increased from 2.13 in the control to values exceeding 11.0 in the 60–90% CP range (Table 3). No statistically significant differences were found among these levels, indicating that yellowness plateaued at approximately 60% CP. A significant decrease was recorded at 100% CP (9.88), with the value comparable to that of the 50%WF/50%CP mixture (9.65), indicating a nonlinear relationship between CP content and perceived yellowness (Table 3).

The observed changes in color parameters are primarily attributed to the presence of natural pigments in the insect material, including melanins, carotenoids, and phenolic compounds [38,39]. Due to melanin-rich cuticle components, the progressive decrease in L* values reflects the inherently darker color of cricket powder. The increase in a* and b* values at intermediate substitution levels may be associated with improved visibility of reddish and yellow pigments when combined with the lighter wheat flour, which promotes light scattering. In contrast, the lower b* value at 100% CP likely results from the optical dominance of dark pigments, which strongly absorb light and reduce the perception of brighter tones, particularly yellow. These findings suggest that color perception depends on pigment concentration and the proportions and optical interactions of the individual ingredients.

The addition of powdered insects imparted a characteristic brownish coloration to the mixtures, resembling the appearance of flours used in wholegrain products [40,41]. Cecchi et al., 2019 [42] reported that consumers often perceive the darker color of fortified products as a typical feature of “healthy” foods with high fiber content.

### 3.2. Particle Size Distribution

Particle size distribution is influenced by wheat variety, milling method, and intended end use [43,44]. For commercial wheat flour, the preferred particle size is generally much finer than 250–350 μm, with most high-quality flours for bread, noodles, and general baking falling below 250 μm [43,44]. A particle size in the 125–180 μm range is widely recognized as medium for wheat and other cereal flours and is frequently used in research on flour quality, dough properties, and baking performance [45]. Wheat flour with a medium particle size (125–180 μm) supports the formation of a gluten network capable of retaining fermentation gases, promoting proper dough expansion and favorable loaf volume, while avoiding excessive crumb firmness or shaping difficulties.

Insect powders typically have a much larger and more variable particle size than wheat flour [46]. According to the European Food Safety Authority (2024) [47], powdered *Acheta domesticus* contains particles ranging from 0.18 to 1.5 mm, indicating a substantially coarser and more heterogeneous structure [47].

The particle sizes of the investigated samples measured by sieving are reported in Table 4.

Based on the results presented in Table 4, wheat flour was characterized mainly by particles of 180 μm, followed by the 125 μm fraction. In contrast, cricket powder exhibited a distribution skewed towards coarser particles, with the 500 μm fraction being the most abundant.

Increasing the proportion of cricket powder in wheat flour blends resulted in statistically significant (*p* < 0.05) shifts in the shares of the 500 μm, 180 μm, and 125 μm fractions compared with the control (100% WF), with significant changes observed at the 15% CP level. The proportion of the 500 μm fraction increased systematically with higher CP content, while the 180 μm and 125 μm fractions decreased. Particles < 125 μm occurred only in trace amounts in all samples, with no significant differences between variants.

These results confirm that the incorporation of cricket powder shifts the particle size distribution towards coarser fractions, consistent with its naturally larger and more heterogeneous particle profile [43,44,45]. At CP levels above 15–20%, the marked reduction in the 180 μm fraction and increase in the ≥500 μm fraction may hinder gluten network formation and reduce dough homogeneity. Coarser particles hydrate more slowly and can disrupt the gluten structure, limiting gas retention and negatively impacting bread texture. From a technological standpoint, maintaining CP inclusion below approximately 10–15% preserves a particle size distribution comparable to wheat flour, helping to retain desirable dough properties and bread quality.

### 3.3. Assessment of Fermentative Properties

Blends of wheat flour with *Acheta domesticus* flour exhibited significant differences in fermentation performance compared to the control sample (100% wheat flour), as confirmed by measurements of maximum dough volume, the distribution characteristics of CO_2_ bubbles, dough collapse time, and the assessment of rise uniformity. The results are presented in Table 5.

The study results showed that wheat flour blends with cricket powder differed in their fermentation performance, assessed based on maximum dough volume, distribution, and characteristics of CO_2_ bubbles, collapse time, and uniformity of rise (Table 5).

The control sample (100% wheat flour) was characterized by the highest mean maximum volume (250 mL), numerous evenly distributed bubbles, and a stable rise lasting approximately 70–80 min. The addition of insect flour, even at the 5% level, significantly reduced the mean maximum volume (to around 205 mL); however, at 5–15% substitution, relatively uniform CO_2_ bubble distribution and a rise time exceeding 60 min were maintained.

Blends containing 10–15% CP exhibited fewer bubbles, with greater size variability and a tendency to accumulate in the upper layers of the sample. At the same time, the uniformity of rise was already assessed as “less uniform”. The maximum volume of these blends (200–202 mL) and the time of maintaining the maximum volume (approximately 60–70 min) suggest that in this range, the proportion of insect flour still allows dough production with acceptable fermentation properties.

In the variants with 20–25% insect powder, the maximum volume decreased to about 170–180 mL, and the bubble structure became irregular, with larger gas cells concentrated in the upper layers and a compact lower part. The time of maintaining maximum volume in these samples was reduced to 50–55 min, and the uniformity of rise was assessed as “clearly irregular”.

At insect flour levels ≥ 30%, further reductions in maximum dough volume were observed (below 170 mL), along with shorter stability times (approximately 45 min or less) and a lack of uniformity in rise, with isolated large bubbles located mainly in the upper parts of the sample.

The observed changes in fermentation performance of wheat flour–cricket powder (WF–CP) blends are consistent with the particle size distribution patterns reported in Table 4. Wheat flour was characterized by a predominance of particles in the 180 μm fraction, followed by the 125 μm fraction, both of which are associated with optimal dough structure and gas retention capacity. The gradual replacement of WF with CP at levels ≥ 15% led to statistically significant (*p* < 0.05) shifts in the granulometric profile, notably an increase in the proportion of coarse particles (≥500 μm) and a reduction in the 180 μm and 125 μm fractions. These granulometric changes correspond to the progressive decline in fermentation capacity in Table 5. Blends containing ≤10% CP maintained relatively uniform formation and distribution of CO_2_ gas bubbles, similar to the control (100% WF). However, at CP levels of 15–20%, the gas cells became less uniformly distributed, with a tendency to accumulate in the upper regions of the dough matrix. This effect intensified at ≥25% CP, with smaller and more irregularly distributed bubbles in the lower layers and large or single gas bubbles in the upper layers. Such heterogeneity reflects impaired gluten network development and reduced gas retention capacity, most likely resulting from the interference of coarse insect powder particles with gluten matrix formation.

### 3.4. Baking Loss and Crumb Hardness Assessment

#### 3.4.1. Baking Loss

Typical baking loss for wheat bread ranges from about 10% to 15%, but can vary depending on recipe, flour type, and baking method [26,48,49]. Our results (Table 6) fall within this range, as the conventionally baked wheat breads demonstrated a baking loss of 13.75%, according to other studies on conventional bread baking at oven temperatures ranging from 180 to 220 °C [50,51]. In traditional baking, the weight loss of bread results solely from water evaporation, as complete crust formation prevents any dough residues from remaining in the baking chamber [49].

Adding cricket powders at up to 15% levels did not cause a significant increase in baking loss, while higher proportions impaired the bread structure (Table 6).

#### 3.4.2. Crumb Hardness Assessment

Hardness is widely recognized as one of the key parameters in evaluating bread texture. It is influenced by multiple factors, including the type of additives used, the dough preparation method, and the mass of the crumb, all of which significantly affect consumer acceptance [52].

In the present study, adding insect powder significantly affected the crumb hardness (Table 6). At a 5–10% inclusion level of insect flour in a blend with type 750 wheat flour, hardness values were comparable to the control sample (17.70–18.10 mm). This observation is consistent with scientific reports indicating that such substitution levels allow sufficient gluten development to maintain bread texture and structure [4].

At levels ≥ 15%, penetration values decreased markedly (e.g., 11.45 mm for 15% CP), indicating a substantial increase in crumb hardness (Table 6). This effect can be explained by the partial replacement of starch and gluten in wheat flour with insect protein and fat—ingredients that are both non-starch and non-gluten—which interferes with gluten network formation and reduces gas retention during fermentation, ultimately leading to a denser crumb structure [7,11,53,54].

A very high proportion of insect powder (from 70% in wheat flour) led to structural instability and difficulties in measurement (Table 6).

#### 3.4.3. Visual Appearance of Bread and Crumb

To illustrate the effect of cricket powder level on the visual characteristics of bread and crumb structure, photographic documentation of loaves and their cross-sections was prepared (Table 7).

### 3.5. Sensory Assessment

The inclusion of insect powder (*Acheta domesticus*) in wheat flour blends had a significant effect (*p* < 0.05) on all analyzed sensory attributes of bread (Table 8). As the proportion of insect powder increased, a systematic decrease in scores was observed compared with the control sample (100% wheat flour, no insect powder). A significant reduction in scores was noted at the following insect powder inclusion levels: overall assessment and flavor—from 10%, taste—from 20%, hardness—from 25%, chewiness—from 35%, and gumminess—from 40%.

The overall assessment of the control sample containing 100% wheat flour was 7.67 points, corresponding to a level between “like moderately” (7) and “like very much” (8) on the 9-point hedonic scale. Adding up to 15% of insect powder did not lower the score below 6.0 points, corresponding to the “like slightly” category. At 20% and 25% inclusion levels, the scores were 5.73 and 5.67 points, respectively, which fall between “like slightly” (6) and “neither like nor dislike” (5). An inclusion level of 35% resulted in a score of 5.00, corresponding to the “neither like nor dislike” category. Increasing the insect powder content to 40% or more reduced the overall score below 5.0 points, indicating a shift to the “dislike slightly” (4) category or lower, and thus a loss of sensory acceptability. The lowest score was recorded for the variant containing 100% cricket powder (100% CP)—1.67 points, corresponding to the “dislike extremely” (1) category (Table 2).

Pearson’s correlation coefficient analysis (Table 9) showed that the overall assessment was most strongly determined by taste (r = 0.87; *p* < 0.0001), flavor (r = 0.82; *p* < 0.0001), and chewiness (r = 0.81; *p* < 0.0001).

The results of the sensory evaluation confirm that the inclusion of insect powder in bread formulations has a significant impact on consumer acceptability, with flavor-related attributes and texture properties—particularly chewiness—and, to a lesser extent, hardness and gumminess, playing a key role in shaping the overall assessment (Table 9). The strongest correlation with overall assessment was found for taste (r = 0.87), consistent with previous reports indicating that taste is the primary determinant of the acceptability of bakery products, regardless of the type of raw material used [55,56,57].

The reduction in sensory acceptability in samples with a high proportion of insect powder was expected. Consumer acceptance tends to decrease as insect powder inclusion increases, mainly due to the intensification of characteristic flavors and aromas [58,59,60].

Studies show that while low levels of insect powder can impart pleasant nutty or roasted notes, higher concentrations often result in more pronounced earthy or off-flavors, which many consumers find undesirable [58,59,60]. This earthy aroma is particularly characteristic of *Acheta domesticus*, where sensory evaluations consistently identify it as a dominant feature [61].

In the context of texture attributes, chewiness was found to be the third most influential factor on overall assessment after taste and flavor (r = 0.81) (Table 9). The decrease in this attribute at high insect powder inclusion levels may result from reduced gluten content in the blend, which limits the dough’s gas retention capacity and affects crumb structure [62,63].

At the same time, the increase in non-starch fractions, including chitin, may have enhanced the perception of gumminess, as confirmed by the correlation results for gumminess (r = 0.72). Current research indicates that increasing non-starch fractions, particularly chitin, in insect bread formulations enhances gumminess by disrupting the gluten–starch matrix and altering bread texture. While enzymatic treatments may mitigate some adverse effects, the overall impact of chitin is an increase in gumminess and a decrease in consumer acceptability [64,65].

The threshold of practical sensory acceptability, determined in our study at 20–25% cricket powder, is consistent with the findings of Mafu et al., 2022 [24]. These authors demonstrated that the overall acceptability of bread enriched with 10% house cricket flour did not differ significantly (*p* < 0.05) from bread enriched with 20% cricket flour. Higher inclusions (≥30%) resulted in a marked decrease in acceptability among consumers unaccustomed to insect-based products [60]. Similar trends were observed in other baked goods (pancakes), where increasing the cricket content to 30% led to the lowest acceptability scores, with flavor being the primary driver of rejection [66].

In summary, our findings confirm that adding insect powder in bread at levels of up to 15% is acceptable to consumers regarding sensory attributes, while a 20–25% inclusion is at the borderline of acceptability. It is considered unacceptable for bread that has over 35% insect powder. In light of the available literature, lower inclusion ranges should be used for the general population. At the same time, sensory acceptance may be improved through consumer education, appropriate flavor-masking techniques, and texture enhancement. For example, sourdough fermentation of insect flours has been reported to improve flavor profiles and increase consumer liking [67,68]. Moreover, adding herbs and spices can reduce the characteristic taste of insects [69]. Textural properties, in turn, may benefit from technological approaches such as incorporating hydrocolloids (e.g., β-glucans, gums) [70], applying specific enzymes [71], or optimizing dough hydration to promote the development of a stable gluten network [72].

### 3.6. Nutritional Value and Nutritional Quality Index

Wheat flour (WF) and insect powder from *Acheta domesticus* differ significantly in their chemical composition. WF is composed mainly of carbohydrates (68.00 g/100 g), including starch (66.60 g/100 g), with a moderate protein content (11.60 g/100 g) and a low fat content (1.80 g/100 g) (Table 10). It contains low amounts of vitamins and minerals, translating into low INQ values for protein and micronutrients about the recommended daily intake (Table 11, Table 12, Table 13 and Table 14).

Insect powder is characterized by a very high protein content (70.00 g/100 g) and fat content (22.80 g/100 g), with minimal carbohydrates (0.50 g/100 g) and no starch. Compared with wheat flour, it contains much higher amounts of dietary fiber (9.50 g/100 g), zinc (18.64 mg/100 g), and riboflavin (11.07 mg/100 g) (Table 10, Table 11 and Table 12). The high INQ values for these nutrients (protein = 6.32; zinc = 10.52; riboflavin = 45.43) (Table 10, Table 13 and Table 14) indicate that insect powder provides them in large amounts relative to its energy content, which substantially increases the nutrient density of products and is beneficial from the perspective of dietary balance.

Blends of WF with added CP showed a proportional increase in protein, fat, fiber, vitamins, and minerals and decreased in carbohydrates and starch with increasing CP content (Table 10, Table 11 and Table 12). Sensory evaluation indicated that 15% CP was the maximum level at which full consumer acceptability was maintained (Table 8). At this level, protein, zinc, iron, and riboflavin contents were 20.36 g/100 g, 3.95 mg, 2.30 mg, and 1.73 mg, respectively (Table 10, Table 11 and Table 12), with INQ values of 2.37, 2.88, 0.74, and 1.73 (Table 13 and Table 14). This level represented an optimal compromise between improving nutritional value and maintaining the desirable sensory properties of bread.

Further increasing the CP content, despite continuing to improve nutritional value (e.g., at 30% CP protein content reached 29.12 g/100 g, zinc 6.54 mg, iron 3.00 mg, and riboflavin 3.38 mg), was associated with decreased scores across all sensory attributes (Table 8) and deterioration of crumb structure, likely related to the reduction in gluten and starch contents, which are responsible for dough rheological properties.

These findings confirm the need for a balanced approach to enriching bakery products with insect powder to maximize nutritional benefits while maintaining consumer acceptance. Nevertheless, applying targeted technological and formulation strategies may allow for higher levels of insect powder incorporation without compromising sensory quality. Implementing such solutions could make it possible to exceed the 15% addition level, further enhancing bakery products’ nutritional value while preserving consumer acceptability.

## 4. Conclusions

The gradual replacement of wheat flour (WF) with *Acheta domesticus* powder (CP) significantly influenced bread quality, with effects dependent on the substitution level. Up to 10–15% CP maintained acceptable fermentation stability (maximum dough volume > 200 mL; rise time ≥ 60 min), uniform crumb porosity, and favorable sensory scores, while substantially increasing protein content and INQ values for selected vitamins and minerals. At ≥20% CP, loaf volume and fermentation stability declined markedly, crumb porosity became irregular, and hardness increased, reducing consumer acceptance. Particle size analysis revealed a progressive increase in coarse fractions (≥500 μm) and a decrease in optimal 125–180 μm particles with increasing CP, likely impairing the gluten network’s gas-holding capacity and contributing to structural defects. These results suggest that CP inclusion should be limited to ≤15% in standard wheat bread formulations to balance nutritional enhancement with technological and sensory quality.

### Practical Recommendations

Although a 10–15% inclusion rate of *Acheta domesticus* powder appears optimal for commercial wheat bread production, large-scale implementation requires consideration of several factors. Insect powders are more expensive than conventional protein ingredients, but economies of scale and increased production are expected to reduce costs. From a regulatory perspective, *Acheta domesticus* powder is already authorized as a novel food ingredient under Regulation (EU) 2015/2283 [14]; however, its use in bakery products requires strict adherence to labeling and maximum inclusion standards. Consumer acceptance also plays a crucial role: our findings indicate that sensory quality is maintained at ≤15% inclusion, yet successful market adoption will depend on effective education campaigns emphasizing sustainability, nutritional benefits, and food safety. These aspects indicate that the successful application of cricket powder requires technological optimization, economic viability, regulatory compliance, and proactive consumer communication. At higher inclusion levels, formulation adjustments become necessary, such as particle size reduction in cricket powder, dough hydration, and mixing time optimization, or using gluten-strengthening additives to mitigate structural weakening and maintain loaf quality. Finally, it should be noted that cricket proteins may trigger allergic reactions, particularly in individuals allergic to crustaceans or dust mites; therefore, potential allergenicity must be considered a consumer safety issue and clearly indicated in product labeling.

## Figures and Tables

**Table 1:** Recipe for bread variants with wheat flour (WF) and cricket powder (CP).

Ingredients	WF	M1	M2	M3	M4	M5	M6	M7	M8	M9	M10	M11	M12	M13	CP
WFr (%)	100	95	90	85	80	75	70	65	60	50	40	30	20	10	-
Yeast (g)	6	6	6	6	6	6	6	6	6	6	6	6	6	6	6
Oil (g)	12	12	12	12	12	12	12	12	12	12	12	12	12	12	12
Sugar (g)	10	10	10	10	10	10	10	10	10	10	10	10	10	10	10
Salt (g)	3	3	3	3	3	3	3	3	3	3	3	3	3	3	3
Water (g)	200	200	200	200	200	200	200	200	200	200	200	200	200	200	300
CP (%)	-	5	10	15	20	25	30	35	40	50	60	70	80	90	100

WF—control sample (wheat flour); The content of wheat flour in the control sample was 200 g (100%); M1–M13—mixtures with varying amounts of wheat flour and powdered insects; CP—criket powder.

**Table 2:** 9-point hedonic scale for bread evaluation.

Scale Point	Meaning
1	dislike extremely
2	dislike very much
3	dislike moderately
4	dislike slightly
5	neither like nor dislike
6	like slightly
7	like moderately
8	like very much
9	like extremely

**Table 3:** Colorimetric parameters (L*, a*, b*) of wheat flour (WF), cricket powder (CP), and their mixtures.

Sample Type	L*	a*	b*
100% WF	96.83 ^a^ ± 0.23	−0.07 ^a^ ± 0.03	2.13 ^a^ ± 0.10
95% WF/5% CP	89.24 ^b^ ± 0.55	−0.14 ^b^± 0.02	8.10 ^b^ ± 0.09
90% WF/10% CP	85.69 ^c^ ± 0.79	−0.46 ^c^ ± 0.02	8.35 ^c^ ± 0.05
85% WF/15% CP	84.52 ^c^ ± 0.81	0.07 ^d^ ± 0.03	8.32 ^c^ ± 0.12
80% WF/20% CP	80.31 ^d^ ± 0.99	0.43 ^e^ ± 0.02	8.58 ^cd^ ± 0.05
75% WF/25% CP	80.71 ^d^ ± 0.34	0.64 ^f^ ± 0.02	8.68 ^d^ ± 0.14
70% WF/30% CP	74.90 ^e^ ± 0.43	1.13 ^g^ ± 0.01	9.17 ^e^ ± 0.05
65% WF/35% CP	73.93 ^e^ ± 0.68	1.19 ^gh^ ± 0.02	9.16 ^e^ ± 0.10
60% WF/40% CP	70.57 ^f^ ± 0.82	1.23 ^h^ ± 0.05	9.37 ^f^ ± 0.14
50% WF/50% CP	65.76 ^g^ ± 0.46	1.43 ^i^ ± 0.02	9.65 ^g^ ± 0.03
40% WF/60% CP	61.05 ^h^ ± 0.56	2.04 ^j^ ± 0.04	10.78 ^h^ ± 0.01
30% WF/70% CP	58.46 ^i^ ± 0.34	2.22 ^k^ ± 0.05	11.26 ^i^ ± 0.09
20% WF/80% CP	55.22 ^j^ ± 0.37	2.39 ^l^ ± 0.01	11.57 ^i^ ± 0.04
10% WF/90% CP	52.31 ^k^ ± 0.20	2.38 ^l^ ± 0.07	11.44 ^i^ ± 0.14
100% CP	51.92 ^k^ ± 0.25	2.60 ^m^ ± 0.03	9.88 ^g^ ± 0.06

Results expressed as mean ± SD (n = 3). Values with different uppercase letter within the same column are significantly different (*p* ≤ 0.05).

**Table 4:** Particle size distribution flour (WF), cricket powder (CP), and their mixtures.

Sample Type	500 μm	180 μm	125 μm	<125 μm
100% WF	10 ^a^ ± 1	65 ^a^ ± 1	20 ^a^ ± 1	5 ± 1
95% WF/5% CP	12 ^a^ ± 1	65 ^a^ ± 1	22 ^ab^ ± 1	1 ± 1
90% WF/10% CP	13 ^a^ ± 1	67 ^a^ ± 1	20 ^a^ ± 1	0 ± 0
85% WF/15% CP	20 ^b^ ± 2	55 ^b^ ± 1	25 ^b^ ± 2	0 ± 0
80% WF/20% CP	28 ^c^ ± 2	50 ^b^ ± 2	22 ^ab^ ± 2	0 ± 0
75% WF/25% CP	30 ^c^ ± 2	51 ^b^ ± 2	18 ^a^ ± 2	1 ± 1
70% WF/30% CP	38 ^d^ ± 2	36 ^c^ ± 2	24 ^b^ ± 2	2 ± 1
65% WF/35% CP	40 ^d^ ± 2	35 ^c^ ± 2	25 ^b^ ± 2	0 ± 0
60% WF/40% CP	56 ^e^ ± 3	21 ^d^ ± 2	23 ^ab^ ± 2	0 ± 0
50% WF/50% CP	74 ^f^ ± 4	24 ^d^ ± 2	2 ^c^ ± 1	0 ± 0
40% WF/60% CP	74 ^f^ ± 4	24 ^d^ ± 2	2 ^c^ ± 1	0 ± 0
30% WF/70% CP	77 ^f^ ± 3	18 ^e^ ± 3	5 ^c^ ± 2	0 ± 0
20% WF/80% CP	84 ^g^ ± 4	14 ^e^ ± 3	2 ^c^ ± 1	0 ± 0
10% WF/90% CP	82 ^g^ ± 4	16 ^e^ ± 2	2 ^c^ ± 1	0 ± 0
100% CP	84 ^g^ ± 4	14 ^e^ ± 3	2 ^c^ ± 1	0 ± 0

Results expressed as mean ± SD (n = 3). Different letters in the same column indicate statistically significant differences (*p* < 0.05).

**Table 5:** Assessment of fermentative properties of dough prepared from wheat flour (WF), cricket powder (CP), and their mixtures.

WF/CP [%]	Mean Maximum Volume [mL] ± SD	Formation and Distribution of CO_2_ Bubbles	Collapse Time After Reaching Maximum Volume [min.]	Uniformity of Growth
100/0	250.0 ^a^ ± 4.2	Numerous, similar-sized, progressively enlarging during fermentation, evenly distributed throughout the sample volume	~70–80	Uniform
95/5	205.0 ^b^ ± 7.7	Fewer in number, similar-sized, evenly distributed throughout the sample volume	~70	Uniform
90/10	200.0 ^b^ ± 6.4	Fewer in number, more variable in size, tending to accumulate in the upper layers of the sample	~65–70	Less uniform
85/15	202.5 ^b^ ± 5.6	Fewer in number, more variable in size, tending to accumulate in the upper layers of the sample	~60–65	Less uniform
80/20	180.5 ^c^ ± 2.9	Smaller CO_2_ bubbles, irregular distribution; in the lower layers, noticeably fewer and a dense, non-porous structure, while the upper part showed a concentration of larger bubbles	~50–55	Clearly irregular
75/25	170.0 ^c^ ± 2.9	Smaller bubbles, irregular distribution; lower layers with fewer gas bubbles, dense crumb, upper part with larger bubbles	~50–55	Clearly irregular
70/30	170.0 ^c^ ± 2.3	Small, single CO_2_ bubbles concentrated in the upper part of the sample	~45–50	Highly irregular
65/35	172.5 ^c^ ± 7.2	Fewer CO_2_ bubbles, mainly in the upper part of the sample	~50	Highly irregular
60/40	175.0 ^c^± 5.6	Small, sparse, irregularly distributed CO_2_ bubbles, mainly in the upper part of the sample	~50	Highly irregular
50/50	147.5 ^d^ ± 2.1	Small CO_2_ bubbles, irregular distribution	~40–45	Highly irregular
40/60	135.5 ^e^ ± 6.6	Single, irregular CO_2_ bubbles in the upper part of the sample	~30–35	No uniformity
30/70	115.0 ^f^ ± 7.8	Single, irregular CO_2_ bubbles, mainly in the upper part of the sample	~25–30	No uniformity
20/80	110.0 ^f^ ± 7.0	Single, irregular CO_2_ bubbles, mainly in the upper part of the sample	~20–25	No uniformity
10/90	110.0 ^f^ ± 3.3	Single, irregular CO_2_ bubbles, mainly in the upper part of the sample	~20	No uniformity
0/100	105.0 ^f^ ± 3.1	Single, irregular CO_2_ bubbles	~20	No uniformity

Different letters in the same column indicate statistically significant differences (*p* < 0.05).

**Table 6:** Baking loss and crumb hardness of breads formulated with wheat flour (WF), cricket powder (CP), and WF–CP blends.

Flours and Flour Blends	Baking Loss [%]	Hardness [mm]
100%WF	13.75 ^a^ ± 0.11	18.30 ^a^ ± 1.50
95%WF/5%CP	14.03 ^a^ ± 0.12	18.10 ^a^ ± 0.42
90%WF/10%CP	13.89 ^a^ ± 0.28	17.70 ^a^ ± 0.28
85%WF/15%CP	14.04 ^a^ ±0.11	11.45 ^b^ ± 0.35
80%WF/20%CP	16.32 ^b^ ± 0.15	11.85 ^b^ ± 0.10
75%WF/25%CP	17.25 ^c^ ± 0.60	11.95 ^b^ ± 0.21
70%WF/30%CP	17.70 ^cd^ ± 0.50	10.90 ^b^ ± 0.14
65%WF/35%CP	18.15 ^de^ ± 0.26	10.20 ^b^ ± 0.28
60%WF/40%CP	18.58 ^e^ ± 0.62	11.00 ^b^ ± 0.28
50%WF/50%CP	19.94 ^f^ ± 0.40	10.70 ^b^ ± 0.26
40%WF/60%CP	20.00 ^f^ ± 0.17	10.15 ^b^ ± 0.10
30%WF/70%CP	20.11 ^f^ ± 0.15	✕
20%WF/80%CP	20.30 ^f^ ±0.36	✕
10%WF/90%CP	20.22 ^f^ ± 0.22	✕
100%CP	22.34 ^g^ ± 0.12	✕

Results expressed as mean ± SD (n = 3). Different letters in the same column indicate statistically significant differences (*p* < 0.05); WF—wheat flour; CP—cricket powder; ✕—the determination could not be performed.

**Table 7:** Visual appearance of bread and crumb prepared from mixtures of wheat flour (WF) and cricket powder (CP) in the range from 0% to 100%.

	100% WF	95%WF/ 5%CP	90%WF/ 10%CP	85%WF/ 15%CP	80%WF/ 20%CP	75%WF/ 25%CP	70%WF 30%CP	65%WF/ 35%CP
Bred	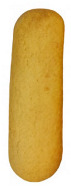	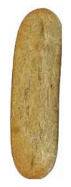	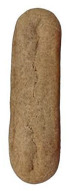	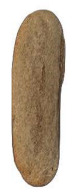	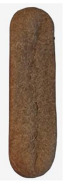	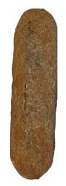	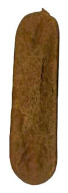	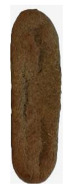
Crumb	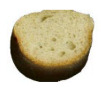	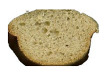	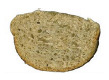	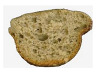	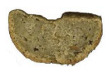	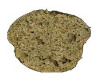	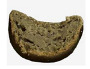	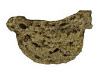
	60%WF/ 40%CP	50%WF/ 50%CP	40%WF/ 60%CP	30%WF/ 70%CP	20%WF/ 80%CP	10%WF/ 90%CP	100%CP	
Bred	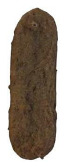	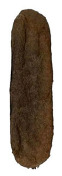	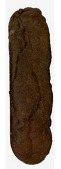	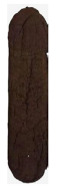	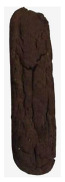	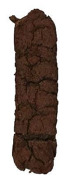	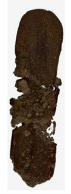	
Crumb	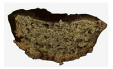	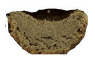	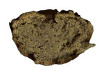	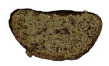	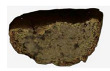	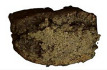	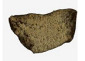	

**Table 8:** Sensory evaluation scores of breads prepared from wheat flour (WF), cricket powder (CP), and their mixtures.

Sample Type	Taste	Flavor	Hardness	Chewing	Gumminess	Overall Assessment
100%WF	7.67 ^a^ ± 0.72	7.73 ^a^ ± 1.39	6.47 ^a^ ± 1.96	6.53 ^a^ ± 2.10	6.00 ^a^ ± 1.93	7.67 ^a^ ± 0.82
95%WF/5%CP	7.67 ^a^ ± 0.72	6.87 ^ab^ ± 1.36	5.60 ^ab^ ± 1.88	6.27 ^ab^ ± 1.44	5.73 ^ab^ ± 1.67	6.73 ^ab^ ± 1.22
90%WF/10%CP	7.40 ^a^ ± 1.12	6.40 ^b^ ± 1.35	5.47 ^ab^ ± 1.77	6.13 ^ab^ ± 1.73	5.60 ^ab^ ± 1.35	6.47 ^bc^ ± 1.30
85%WF/15%CP	6.73 ^ab^ ± 1.16	6.20 ^b^ ± 1.42	5.40 ^ab^ ± 1.72	5.67 ^abc^ ± 1.84	5.40 ^ab^ ± 1.64	6.27 ^bcd^ ± 1.44
80%WF/20%CP	6.40 ^bc^ ± 0.99	6.07 ^bc^ ± 1.03	5.40 ^ab^ ± 0.99	5.60 ^abc^ ± 1.64	5.40 ^ab^ ± 1.30	5.73 ^bcde^ ± 1.16
75%WF/25%CP	6.20 ^bc^ ± 0.86	6.07 ^bc^ ± 1.87	5.13 ^b^ ± 1.06	5.53 ^abc^ ± 1.30	5.33 ^ab^ ± 1.45	5.67 ^cde^ ± 1.23
70%WF/30%CP	6.13 ^bc^ ± 1.30	6.00 ^bc^ ± 1.85	5.13 ^b^ ± 1.73	5.53 ^abc^ ± 1.82	5.00 ^ab^ ± 1.36	5.33 ^def^ ± 1.63
65%WF/35%CP	5.60 ^cd^ ± 0.99	6.00 ^bc^ ± 1.00	5.07 ^b^ ± 0.96	5.00 ^bcd^ ± 1.20	4.80 ^ab^ ± 1.37	5.00 ^efg^ ± 1.00
60%WF/40%CP	5.47 ^cd^ ± 1.30	5.93 ^bc^ ± 1.79	4.87 ^b^ ± 1.30	5.00 ^bcd^ ± 1.20	4.73 ^b^ ± 1.03	4.93 ^efg^ ± 0.88
50%WF/50%CP	4.73 ^de^ ±1.49	4.80 ^cde^ ± 1.82	4.73 ^bc^ ± 1.58	4.40 ^cd^ ± 1.70	4.67 ^b^ ± 1.18	4.60 ^fgh^ ± 1.50
40%WF/60%CP	4.33 ^ef^ ± 1.80	4.33 ^ed^ ± 1.95	4.60 ^bc^ ± 2.03	4.27 ^de^ ± 1.45	4.47 ^b^ ± 1.77	4.27 ^gh^ ± 1.87
30%WF/70%CP	3.47 ^fg^ ± 1.36	4.13 ^ed^ ± 1.60	4.60 ^bc^ ± 2.06	4.27 ^de^ ± 1.71	4.47 ^b^ ± 1.41	3.67 ^hi^ ± 1.35
20%WF/80%CP	2.87 ^gh^ ± 1.51	3.47 ^df^ ± 2.17	3.53 ^cd^ ± 1.77	3.47 ^e^ ± 1.58	3.33 ^c^ ± 1.40	3.20 ^i^ ± 1.21
10%WF/90%CP	2.67 ^gh^ ± 1.45	3.67 ^df^ ± 2.13	2.67 ^de^ ± 1.63	3.40 ^e^ ± 1.55	3.27 ^c^ ± 2.02	3.13 ^i^ ± 1.46
100%CP	2.00 ^h^ ± 1.25	2.73 ^f^ ± 1.94	1.87 ^e^ ± 1.19	1.93 ^f^ ± 1.42	1.73 ^d^ ± 1.16	1.67 ^j^ ± 0.98

Results expressed as mean ± SD (n = 25). WF—wheat flour; CP—cricket powder; Different letters in the same column indicate statistically significant differences (*p* < 0.05).

**Table 9:** Pearson correlation coefficients between sensory characteristics of wheat flour–cricket powder and their mixtures.

	Taste	Flavor	Hardness	Chewiness	Gumminess	Overall Assessment
Taste	1.00	0.74 ***	0.63 ***	0.72 ***	0.67 ***	0.87 ***
Flavor		1.00	0.68 ***	0.74 ***	0.66 ***	0.82 ***
Hardness			1.00	0.73 ***	0.73 ***	0.71 ***
Chewiness				1.00	0.74 ***	0.81 ***
Gumminess					1.00	0.72 ***
Overall assessment						1.00

*** *p* < 0.0001 for all correlation coefficients; significance evaluated at α = 0.05.

**Table 10:** The basic nutrients content of flours and flour blends per 100 g.

Flours and Flour Blends	Energy [kcal/100 g]	Protein [g/100 g]	Fat [g/100 g]	Carbohydrates [g/100 g]	Fiber [g/100 g]	Starch [g/100 g]	INQ Values for Protein
100%WF	342.00	11.60	1.80	68.00	2.90	66.60	1.42
95%WF/5%CP	347.68	14.52	2.64	64.63	3.23	63.27	1.75
90%WF/10%CP	353.35	17.44	3.48	61.25	3.56	59.94	2.06
85%WF/15%CP	359.03	20.36	4.31	57.88	3.89	56.61	2.37
80%WF/20%CP	364.70	23.28	5.15	54.50	4.22	53.28	2.67
75%WF/25%CP	370.38	26.20	5.99	51.13	4.55	49.95	2.96
70%WF/30%CP	376.05	29.12	6.83	47.75	4.88	46.62	3.24
65%WF/35%CP	381.73	32.04	7.66	44.38	5.21	43.29	3.51
60%WF/40%CP	387.40	34.96	8.50	41.00	5.54	39.96	3.77
50%WF/50%CP	398.75	40.80	10.18	34.25	6.20	33.30	4.28
40%WF/60%CP	410.10	46.64	11.85	27.50	6.86	26.64	4.75
30%WF/70%CP	421.45	52.48	13.53	20.75	7.52	19.98	5.21
20%WF/80%CP	432.80	58.32	15.20	14.00	8.18	13.32	5.63
10%WF/90%CP	444.15	64.16	16.88	7.25	8.84	6.66	6.04
100%CP	463.00	70.00	22.80	0.50	9.50	0.00	6.32

WF—wheat flour, CP—cricket powder.

**Table 11:** Mineral contents of flours and flour blends (mg/100 g).

Flours and Flour Blends	Na	K	Ca	P	Mg	Fe	Zn	Cu	Mn
100%WF	3.00	165.00	20.00	122.00	31.00	1.60	1.36	0.15	0.62
95%WF/5%CP	24.63	213.08	25.61	154.90	33.45	1.83	2.22	0.19	0.74
90%WF/10%CP	46.26	261.16	31.21	187.80	35.90	2.07	3.09	0.22	0.86
85%WF/15%CP	67.89	309.24	36.82	220.70	38.35	2.30	3.95	0.26	0.97
80%WF/20%CP	89.52	357.32	42.43	253.60	40.80	2.53	4.82	0.29	1.09
75%WF/25%CP	111.15	405.41	48.04	286.50	43.25	2.77	5.68	0.33	1.21
70%WF/30%CP	132.78	453.49	53.64	319.40	45.70	3.00	6.54	0.36	1.33
65%WF/35%CP	154.41	501.57	59.25	352.30	48.15	3.23	7.41	0.40	1.44
60%WF/40%CP	176.04	549.65	64.86	385.20	50.60	3.47	8.27	0.43	1.56
50%WF/50%CP	219.30	645.81	76.07	451.00	55.50	3.94	10.00	0.50	1.80
40%WF/60%CP	262.56	741.97	87.28	516.80	60.40	4.40	11.73	0.57	2.03
30%WF/70%CP	305.82	838.13	98.50	582.60	65.30	4.87	13.46	0.64	2.27
20%WF/80%CP	349.08	934.30	109.71	648.40	70.20	5.34	15.18	0.71	2.50
10%WF/90%CP	392.34	1030.46	120.93	714.20	75.10	5.80	16.91	0.78	2.74
100%CP	435.60	1126.62	132.14	780.00	80.00	6.27	18.64	0.85	2.97

Na—sodium; K—potassium; Ca—calcium, P—phosphorus; Mg—magnesium; Fe—iron; Zn—zinc; Cu—copper; Mn—manganese; WF—wheat flour, CP—cricket powder.

**Table 12:** Vitamin contents of flours and flour blends per 100 g.

Flours and Flour Blends	Vit. A [µg]	Vit. B_1_ [mg]	Vit. B_2_ [mg]	Vit PP [mg]	Vit. C [mg]	Vit. E [mg]
100%WF	0.00	0.32	0.08	2.29	0.00	0.74
95%WF/5%CP	1.22	0.31	0.63	2.81	0.49	0.92
90%WF/10%CP	2.43	0.30	1.18	3.32	0.97	1.10
85%WF/15%CP	3.65	0.29	1.73	3.84	1.46	1.27
80%WF/20%CP	4.87	0.29	2.28	4.35	1.95	1.45
75%WF/25%CP	6.08	0.28	2.83	4.87	2.44	1.63
70%WF/30%CP	7.30	0.27	3.38	5.38	2.92	1.81
65%WF/35%CP	8.52	0.26	3.93	5.90	3.41	1.98
60%WF/40%CP	9.73	0.25	4.48	6.41	3.90	2.16
50%WF/50%CP	12.17	0.23	5.58	7.44	4.87	2.52
40%WF/60%CP	14.60	0.21	6.67	8.47	5.84	2.87
30%WF/70%CP	17.03	0.19	7.77	9.50	6.82	3.23
20%WF/80%CP	19.46	0.17	8.87	10.53	7.79	3.58
10%WF/90%CP	21.90	0.15	9.97	11.56	8.77	3.94
100%CP	24.33	0.13	11.07	12.59	9.74	4.29

Vit. A—Vitamin A (retinol); Vit. B_1_—Vitamin B_1_ (thiamine); Vit B_2—_Vitamin B_2_ (riboflavin);Vit. PP—Vitamin PP (niacin); Vit. C—Vitamin C (ascorbic acid); Vit. E—Vitamin E (tocopherols); WF—wheat flour, CP—cricket powder.

**Table 13:** INQ values of selected minerals following the demand of women (see Materials and Methods section).

Flours and Flour Blends	INQ Values								
	Na [mg]	K [mg]	Ca [mg]	P [mg]	Mg [mg]	Fe [mg]	Zn [mg]	Cu [mg]	Mn [mg]
100%WF	0.01	0.29	0.12	1.07	0.59	0.54	1.04	1.02	2.10
95%WF/5%CP	0.10	0.37	0.15	1.33	0.63	0.61	1.67	1.24	2.46
90%WF/10%CP	0.18	0.44	0.18	1.59	0.66	0.68	2.28	1.45	2.81
85%WF/15%CP	0.26	0.51	0.21	1.84	0.70	0.74	2.88	1.65	3.15
80%WF/20%CP	0.34	0.59	0.24	2.08	0.73	0.81	3.45	1.85	3.47
75%WF/25%CP	0.42	0.65	0.27	2.31	0.76	0.87	4.01	2.04	3.79
70%WF/30%CP	0.49	0.72	0.30	2.54	0.79	0.93	4.55	2.22	4.09
65%WF/35%CP	0.56	0.78	0.32	2.76	0.82	0.98	5.07	2.40	4.39
60%WF/40%CP	0.63	0.85	0.35	2.97	0.85	1.04	5.58	2.58	4.68
50%WF/50%CP	0.77	0.97	0.40	3.38	0.91	1.15	6.55	2.91	5.23
40%WF/60%CP	0.89	1.08	0.44	3.76	0.96	1.25	7.47	3.23	5.75
30%WF/70%CP	1.01	1.19	0.49	4.13	1.01	1.34	8.34	3.53	6.24
20%WF/80%CP	1.12	1.29	0.53	4.47	1.06	1.43	9.17	3.81	6.71
10%WF/90%CP	1.23	1.39	0.57	4.80	1.10	1.52	9.95	4.08	7.15
100%CP	1.31	1.45	0.60	5.03	1.13	1.57	10.52	4.26	7.45

Column heading abbreviations are as follows: Na—sodium; K—potassium; Ca—calcium, P—phosphorus; Mg—magnesium; Fe—iron; Zn—zinc; Cu—copper; Mn—manganese. WF—wheat flour, CP—cricket powder.

**Table 14:** INQ values of selected vitamins following the demand of women (see Materials and Methods section).

Flours and Flour Blends	INQ Values					
	Vit. A [µg]	Vit. B_1_ [mg]	Vit. B_2_ [mg]	Vit PP [mg]	Vit. C [mg]	Vit. E [mg]
100%WF	0.00	1.80	0.45	1.00	0.00	0.57
95%WF/5%CP	0.01	1.72	3.45	1.20	0.04	0.69
90%WF/10%CP	0.02	1.64	6.34	1.40	0.08	0.81
85%WF/15%CP	0.03	1.56	9.15	1.59	0.11	0.93
80%WF/20%CP	0.04	1.49	11.87	1.78	0.15	1.04
75%WF/25%CP	0.05	1.41	14.51	1.96	0.18	1.15
70%WF/30%CP	0.06	1.34	17.07	2.14	0.22	1.25
65%WF/35%CP	0.07	1.27	19.55	2.31	0.25	1.36
60%WF/40%CP	0.08	1.21	21.96	2.47	0.28	1.46
50%WF/50%CP	0.09	1.08	26.57	2.79	0.34	1.65
40%WF/60%CP	0.11	0.96	30.92	3.08	0.40	1.83
30%WF/70%CP	0.12	0.85	35.04	3.37	0.45	2.00
20%WF/80%CP	0.13	0.74	38.95	3.63	0.50	2.16
10%WF/90%CP	0.15	0.64	42.65	3.89	0.55	2.31
100%CP	0.16	0.53	45.43	4.06	0.59	2.42

Column heading abbreviations are as follows: A—vitamin A, B_1_—thiamin, B_2_—riboflavin, PP—niacin, C—vitamin C, E—vitamin E. WF—wheat flour, CP—cricket powder.

## Data Availability

The original contributions presented in the study are included in the article, further inquiries can be directed to the corresponding author.
